# Role of mucosal immunity and epithelial–vascular barrier in modulating gut homeostasis

**DOI:** 10.1007/s11739-023-03329-1

**Published:** 2023-07-04

**Authors:** Antonio Di Sabatino, Giovanni Santacroce, Carlo Maria Rossi, Giacomo Broglio, Marco Vincenzo Lenti

**Affiliations:** 1 Department of Internal Medicine and Medical Therapeutics, University of Pavia, Pavia, Italy; 2 First Department of Internal Medicine, San Matteo Hospital Foundation, Pavia, Italy; 3 Clinica Medica I, Fondazione IRCCS Policlinico San Matteo, Università di Pavia, Viale Golgi 19, 27100 Pavia, Italy

**Keywords:** Chronic inflammation, Epithelial monolayer, Gut–vascular barrier, Microbiota, Mucosal immunity

## Abstract

The intestinal mucosa represents the most extensive human barrier having a defense function against microbial and food antigens. This barrier is represented externally by a mucus layer, consisting mainly of mucins, antimicrobial peptides, and secretory immunoglobulin A (sIgA), which serves as the first interaction with the intestinal microbiota. Below is placed the epithelial monolayer, comprising enterocytes and specialized cells, such as goblet cells, Paneth cells, enterochromaffin cells, and others, each with a specific protective, endocrine, or immune function. This layer interacts with both the luminal environment and the underlying *lamina propria*, where mucosal immunity processes primarily take place. Specifically, the interaction between the microbiota and an intact mucosal barrier results in the activation of tolerogenic processes, mainly mediated by FOXP3^+^ regulatory T cells, underlying intestinal homeostasis. Conversely, the impairment of the mucosal barrier function, the alteration of the normal luminal microbiota composition (dysbiosis), or the imbalance between pro- and anti-inflammatory mucosal factors may result in inflammation and disease. Another crucial component of the intestinal barrier is the gut–vascular barrier, formed by endothelial cells, pericytes, and glial cells, which regulates the passage of molecules into the bloodstream. The aim of this review is to examine the various components of the intestinal barrier, assessing their interaction with the mucosal immune system, and focus on the immunological processes underlying homeostasis or inflammation.

## Introduction

The gastrointestinal (GI) tract has a wide surface which is constantly in contact with luminal antigenic stimuli; therefore, the GI tract acts as a complex barrier that mainly consists of four layers, namely the epithelial, immunological, and vascular barrier, and the gut microbiota that can functionally be considered as another independent “layer”. These structures also mediate tolerance toward both self- and non-self-antigens and a disruption of the barrier can lead to immune-mediated disorders [1].

The gut mucosal surface is a semi-permeable structure, where tight junctions (TJs) and zonulin mediate the permeability [2]. Interestingly, zonulin is also thought to influence the gut–epithelial barrier’s tolerance and immunity to self- and non-self-antigens, as its altered regulation may be found both in regional and non-regional disorders [3–8].

The epithelial layer of the barrier is composed of a single lining of cells that mediate selective permeability via two main mechanisms, transepithelial/transcellular and paracellular pathways. The first path is enhanced by selective transporters for amino acids, short-chain fatty acids, electrolytes, and sugars, whereas paracellular transport is mediated via tight junctions, adherens junctions, and desmosomes [9–11].

The intestinal mucosal immunity system relies on the action of both cells, such as Paneth cells, epithelial cells, innate lymphoid cells, intraepithelial lymphocytes, and more complex systems such as Peyer’s patches that are responsible for the production of secretory (s)IgA. Interestingly, both actors can be influenced by microbiota–host interactions [12, 13], and the intestinal innate immune system regulates the adaptive immune reactions to the microbiota itself [14].

Another pivotal component of the aforementioned layers is the gut–vascular barrier, which is located on the gut vessel endothelium. Its structure, sharing some similarities with the blood–brain barrier, includes pericytes and enteric glial cells which, with the help of junctional complexes, limit the paracellular passage of molecules in the bloodstream [15–18].

Lastly, intestinal microbiota, namely the symbiotic bacterial populations in the intestinal lumen, can be considered a functional barrier as it modulates immune responses [12–14]. Furthermore, some evidence suggests that the disruption of this delicate homeostatic balance may trigger the development of autoimmune or immune-mediated disorders [19–22].

Starting from these premises, we herein describe the mechanisms and factors implicated in maintenance of the whole gastrointestinal barrier, including epithelial, immunological, vascular, and microbiota-related interactions.

## Methods

In October 2022, we searched Medline (PubMed) using the medical subject heading terms “gut”, “small bowel”, “epithelial barrier”, “vascular barrier”, “microbiome/microbiota”, and “mucosal immunity” for all articles published since database inception. Thousands of papers were found with this search strategy, the majority of which were unrelated to the subject of this review and were not considered. We therefore selected only studies (both non-human and human) exploring gut–vascular alterations, mucosal immunity, intestinal permeability impairment, and microbiota, prioritizing randomized controlled trials, meta-analysis and systematic reviews when available. We also searched the reference lists of key reviews on the topic for additional papers we considered to be relevant.

## Epithelial barrier

**Fig. 1:**
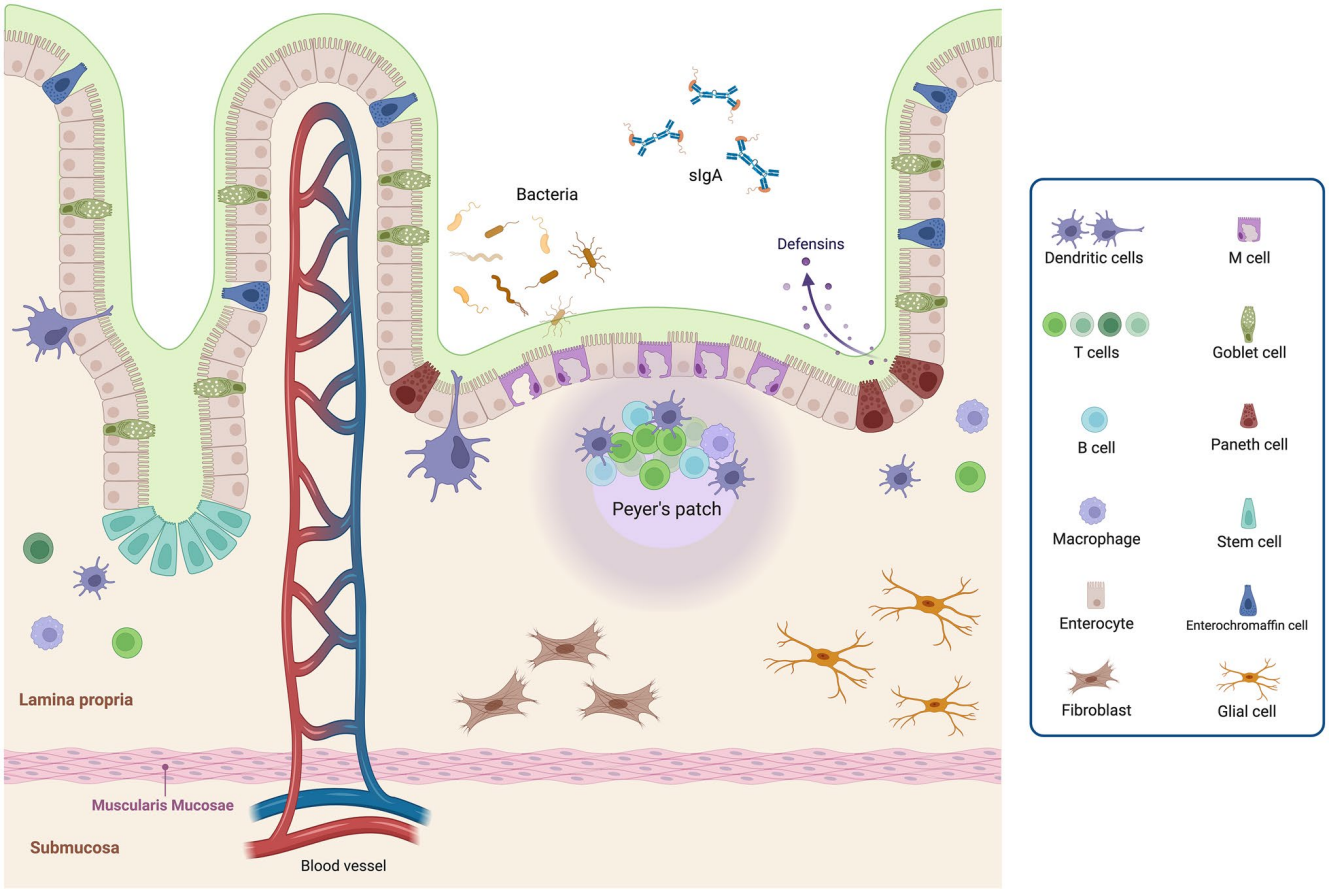
Representation of the main components of the intestinal barrier: epithelial barrier, mucus barrier and lamina propria. The intestinal epithelial monolayer is composed of absorptive enterocytes interspersed with specialized cells: goblet cells, which regulate mucus production; Paneth cells, dedicated to the secretion of anti-microbial peptides; enterochromaffin cells, that are neuroendocrine cells; intestinal stem cells, resident deep within intestinal crypts; dendritic cells, intercalated between epithelial cells for luminal antigens uptake and immune response activation; M cells, underlying Peyer’s plaques facilitating antigens presentation to immune cells. Above the intestinal epithelium, the mucus barrier, composed by mucin, secretory immunoglobulin (sIg)A dimers, and antibacterial peptide (especially defensins), play an antibacterial role, preventing microbial adhesion and invasion. The subepithelial region consists of the lamina propria, composed by (innate and adaptive) immune cells, including lymphoid structures such as Peyer’s patches; network of neurons and glial cells forming the enteric nervous system; connective tissue produced by fibroblasts. Created with “BioRender.com”. *sIgA* secretory immunoglobulin A

The mucous membranes represent one of the main interfaces with the external environment, especially the intestinal mucosa, which consists of a large surface area exposed to continuous contact with numerous dietary and microbial antigens. The intestinal mucosal barrier represents the first line of defense for the body and plays a critical role in modulating the immune tolerance [23, 24]. This barrier consists of an extracellular component, the mucus barrier, the epithelial monolayer (i.e., a layer of enterocytes interspersed with specialized cells), and the underlying *lamina propria*. The physiologic components of the intestinal barrier are schematically represented in Fig. 1.

### Mucus layer

Above the intestinal epithelium, is placed a mucus layer which plays a predominantly antibacterial role, preventing microbial adhesion to the mucosa and subsequent trans-epithelial invasion. This mucus layer has a different composition and properties depending on the intestinal tract under consideration [25, 26]. Notably, the colon, which gets in contact with billions of microorganisms, consists of a protective double-layer system, while the small intestine, which witnesses less contact with bacteria, has a single layer.

In the small intestine, mucus is typically non-attached to epithelial cells and typically covers the villi tips. This single layer consists mainly of the highly glycosylated gel-forming mucin MUC2, produced by goblet cells, mixed with antimicrobial peptides and proteins secreted by both Paneth cells and enterocytes. A proper mucin secretion in the small intestine has been related to the correct function of cellular ion channels. Indeed, in patients with cystic fibrosis, the absence of a functional cystic fibrosis transmembrane regulator (CFTR) and the consequent reduced secretion of bicarbonate, has been related to an adherent, denser, and less penetrable mucus, accounting for the intestinal manifestations of the disease [27].

As mentioned, the large intestine has a double-layer mucus organization system. The outer layer (stirred mucus layer) consists of mucins, predominantly MUC2, sIgA, synthesized at the level of the *lamina propria* [28], and antimicrobial peptides, including defensins that play a role in the setup of the adaptive immune response [29]. The inner and denser layer (non-stirred mucus layer) is strongly attached to the epithelial monolayer and is impermeable to microorganisms. These features are explained by the peculiar organization at this level of MUC2, which forms large net-like structures by N-terminal trimerization and C-terminal dimerization that subsequently assemble into impermeable lamellar networks [30, 31]. The role of MUC2 in defense against microbes has been confirmed in several experimental models in which MUC2 deficiency was correlated with reduced inner mucus layer, resulting in increased intestinal permeability and susceptibility to inflammation and intestinal tumors [32–34]. The inner layer also includes the enterocyte surface glycocalyx, composed by the transmembrane mucins (i.e., MUC3, MUC12, and MUC17) consisting of a cytoplasmic tail, a transmembrane single pass domain and an enormous extracellular mucin domain densely decorated with glycans [35]. The glycocalyx has a protective and structural role, and in addition, its transmembrane mucins would appear to be involved in the process of apical cell surface sensing and signaling [25, 36].

Thus, the intestinal tolerogenic ability, which allows the intestine to be exposed to a large number of microorganisms and foreign food antigens without mounting an inflammatory response, has the mucus barrier as the primary actor involved. This complex layer acts primarily as a physical barrier, but most recent evidence showed a functional mucus modulation of the immune system through tolerogenic signals. In particular, it was shown that MUC2 and its associated glycans can actively modulate, especially via goblet cells, CD103+ dendritic cells (DCs) of the *lamina propria*, conferring a tolerogenic profile [37, 38].

### Epithelial layer

The intestinal epithelial monolayer is composed of absorptive enterocytes interspersed with specialized cells, such as goblet cells, which regulate mucus production [39], Paneth cells, which are dedicated to the secretion of anti-microbial peptides [40], enterochromaffin cells, the most abundant neuroendocrine cells in the gut [41], and intestinal stem cells, that reside deep within intestinal crypts and generate cells that migrate to the upper villi where final differentiation takes place [42].

The epithelial cells that form the monolayer are interconnected and connected to the basement membrane by protein complexes that ensure the structural and functional integrity of the epithelium [23, 43]. The principal types of junctions found in the intestinal epithelium are TJs, adherens junctions (AJs), and desmosomes.

TJs are the intercellular junctions arranged in the most apical region of cell–cell contacts and are the main determinants of epithelial polarity and permeability, regulating the paracellular transport pathway [44]. Such junctions limit the free diffusion of molecules from the cell apex to the basement membrane. The main constituents of TJs are the family of integral membrane proteins called claudins, which link with actin cytoskeleton through the zonula occludens (ZO) family of scaffolding proteins, namely ZO-1 and ZO-2 [45]. Besides claudins, other molecules appear to be determinants of the molecular composition of TJs, including occludin, tricellulin, and junctional adhesion molecules. Although the constituents of these junctions are largely known, how these molecules interact with each other and with the lipid membrane to regulate the intestinal permeability is still a matter of debate. Some cytokines can regulate the function of the TJs barrier through different mechanisms, such as the cytoskeletal modulation. The effects of tumor necrosis factor (TNF), a cytokine with a central pathogenic role in several gastrointestinal diseases, on barrier integrity are the most thoroughly investigated. In particular, TNF has been shown to activate myosin light chain kinase, leading to increase in TJs permeability and subsequent epithelial and endothelial barrier dysregulation [46, 47].

The AJs and desmosomes, placed deeper in the intercellular space, participate in cellular interactions and transepithelial transport regulation. AJs are composed of a family of trans-membrane proteins, called cadherins, which interact with molecules from adjacent cells which link the cytoskeleton. In particular, the direct interaction between E-cadherin, ɑ-catenin, and β-catenin allows the formation of AJs [48]. Desmosomes, made of desmoglein, desmocollin, and desmoplakin, are adhesive junctions that link intermediate filaments [49]. These junctions provide strong adhesive bonds between the cell and cytoskeleton, and their loss results in the disruption of intercellular and cell–matrix contacts, with associated premature Fas–Fas ligand-mediated apoptosis [50].

Evidence accumulated over the years has shown how mucosal barrier is regulated in response to physiological and immunological stimuli and its dysfunction can be associated with the pathogenesis of several intestinal diseases. For example, up-regulation of claudin-2, down-regulation of occludin, and activation of epithelial myosin light chain kinase have been found in the intestinal mucosa of both Crohn's disease and ulcerative colitis patients [51–53]. Similarly, in *Clostridium difficile*-induced colitis, a loss of ZO-1 and ZO-2 was detected [54]. Lastly, absence of phosphorylated ZO-1 and extensive phosphorylation of β-catenin, that would be responsible for disassembly of TJs, were observed in the duodenal mucosa of patients with untreated coeliac disease [55].

Within the epithelium, immune cells may be found. Some of these do not have access to the intestinal lumen, such as intraepithelial lymphocytes, especially those expressing the γδ T receptor, which produce antimicrobial peptides and limit the entrance of commensal bacterial after epithelial injury, thus preserving the host-microbial homeostasis [56, 57]. Others have direct contact with intestinal lumen, such as DCs or neutrophils during infection [58]. DCs, especially those expressing the CXCR1+ chemokine receptor 1 (CXC3CR1), can intercalate between epithelial cells for direct uptake of luminal antigens [59]. These immune cells can behave like macrophages and are implicated in the maintenance of mucosal tolerance through the production of interleukin (IL)-10 [60].

### Lamina propria

The sub-epithelial region consists of the *lamina propria*, composed of immune cells, enteric nervous system (ENS), and connective tissue.

The intestinal *lamina propria* is colonized by effectors of the immune response, specifically the early mentioned CD8+ intraepithelial lymphocytes, *lamina propria* lymphocytes (both B and T cells), eosinophils, DCs, mast cells, and macrophages [39]. Furthermore, gut immune cells are organized to form the so-called gut-associated lymphoid tissue (GALT), which includes lymphoid structures, such as lymphatic follicles, Peyer’s patches, and mesenteric lymph nodes [59]. Of interest is the function of M cells, that constitute the specialized epithelium underlying Peyer’s plaques and with the ability to monitor the intestinal lumen and facilitate the uptake and presentation of luminal antigens to the underlying immune cells [62].

The extracellular matrix of the *lamina propria* is supported by connective tissue produced by fibroblasts. Fibroblasts, in addition to their structural role, actively participate in the epithelial barrier function. Previous studies showed a regulation of epithelial proliferation in response to liver cell growth factor [63], and more recent studies have identified a heterogeneity of fibroblasts with different functions in maintaining homeostasis in the intestine and in responding to tissue damage through secretion of soluble mediators [64]. Specifically, three distinct fibroblast subsets have been recognized. CD81+ fibroblasts maintain the identity and proliferation of intestinal stem cells through the production of WNT ligands, R-spondins, and Gremlin 1. PDGFRα^hi^ fibroblasts mainly regulate the differentiation of cellular epithelium through the production of bone morphogenic proteins and WNT5A. Lastly, PDGFRα(low) fibroblasts secrete basal membrane proteins and contribute to extracellular matrix production and remodeling [65].

Finally, the ENS residing in the *lamina propria*, consists of a network of neurons and glial cells that are organized to form two plexuses, namely the myenteric (Auerbach’s) plexus and the submucosal (Meissner’s) plexus. The ENS represents the effector of the bidirectional interaction between central nervous system and intestine. It is increasingly recognized as a regulator of epithelial barrier integrity and as a potential immunomodulator. Dramatic alterations in the ENS have been demonstrated under conditions of chronic inflammation, such as inflammatory bowel disease, and hence the hypothesis of its pathogenic role in some intestinal diseases [66]. Furthermore, the interaction between enteric microbiota, mucosal immune system, and ENS has been supposed to be involved in the pathophysiology of neurodegenerative disorders, which are often associated with functional gastrointestinal disorders. However, it is still a matter of debate whether changes in the dynamics of this interplay are a consequence of central nervous disorders or may represent the *primum movens* of the neurodegenerative process [67].

## Mucosal immune system

**Fig. 2:**
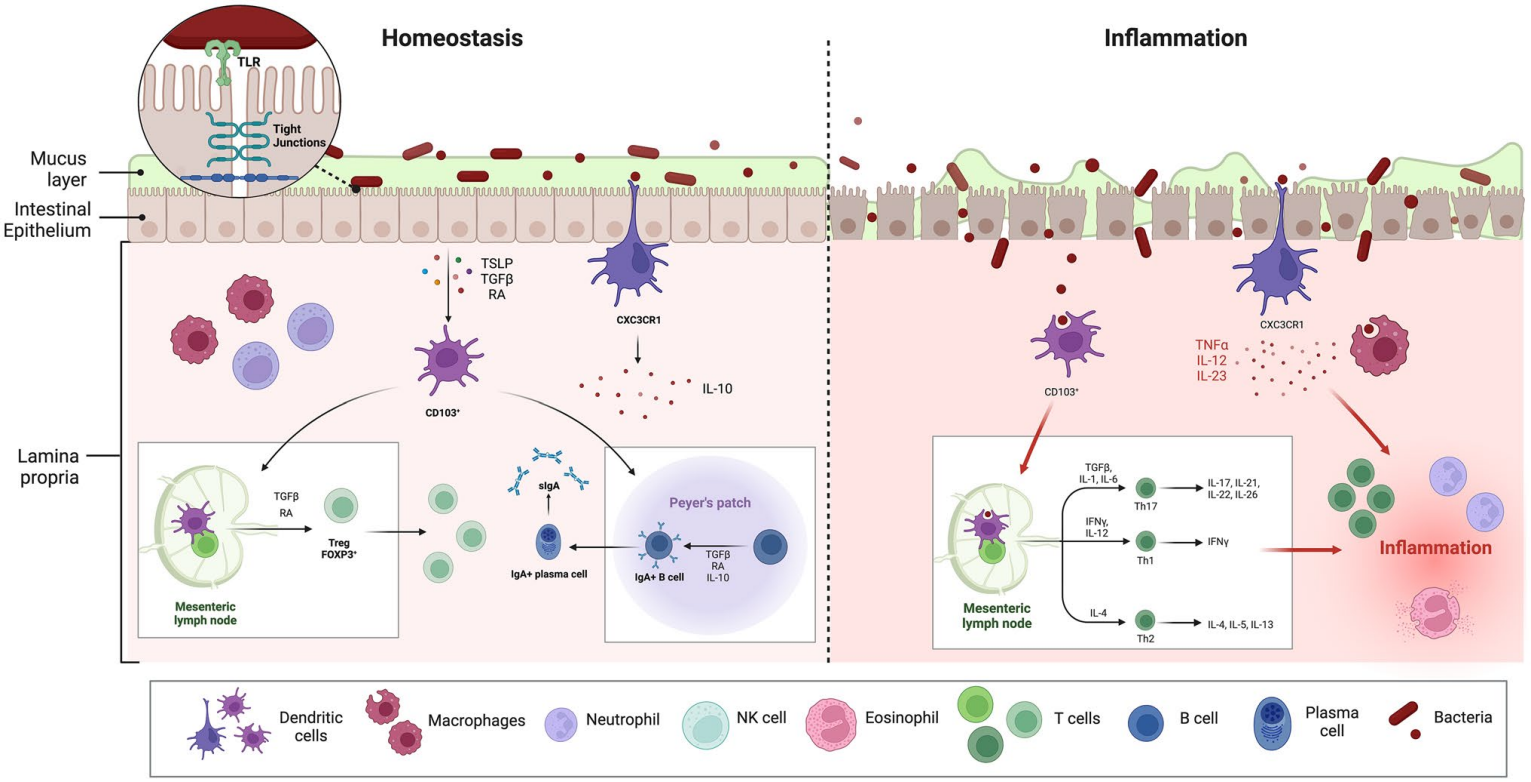
Intestinal homeostasis and inflammation. Mucosal layer and intestinal epithelium act as first barrier against microorganisms and luminal antigens. The intervention of immune system, composed by cells of innate immunity (epithelial cells, dendritic cells (DCs), macrophages and natural killers) and adaptive immunity (B and T cells), is required to mount a specific response. The interaction of epithelial cells (interconnected by junctions) with commensal bacteria, through Toll-Like Receptors, activates a process of tolerance and homeostasis (left of the figure). Specifically, the epithelium produces thymic stromal lymphopoietin, transforming growth factor β, and retinoic acid that stimulate CD103+ DCs to modulate a differentiation toward regulatory T cells (FOXP3+). Also, activated DCs induce maturation of B cells into IgA-secreting plasma cells. CXC3R1 DCs, intercalated between epithelial cells, take up luminal antigens and maintain mucosal tolerance, through the production of IL-10. The impairment of mucus layer and epithelial barrier, associated with dysbiosis, may account for an inflammatory response, leading to disease development (right of the figure). DCs and macrophages, after the interaction with pathogens, direct a differentiation of Th1, Th2, and Th17 cells (also through tumor necrosis factor α, interleukine-12 and -23), and determine the activation of innate immune cells (neutrophils, eosinophils etc.), leading to inflammation. Created with “BioRender.com”. *IL* interleukin, *NK* natural killer, *RA* retinoic acid, *sIgA* secretory immunoglobulin A, *TGF β* transforming growth factor β, *TLR* tool like receptor, *TNF α* tumor necrosis factor, *Treg* regulatory T cell, *TSLP* thymic stromal lymphopoietin

The epithelial barrier, along with mucus and the other protective systems described, is the first barrier to pathogens arising from the intestinal lumen, acting by a nonspecific mechanism. However, the intervention of the immune system is required to mount more specific processes such as the acquisition of tolerance toward non-harmful antigens or the activation of a physiological inflammatory response toward harmful agents. The impairment in the intestinal immune system and in its interaction with gut antigens represents a critical determinant in the development of gut inflammation and allergy (Fig. 2) [68].

The first line of defense is the innate immunity, consisting at the intestinal level of epithelial cells, DCs, macrophages, and natural killer cells. These cells act like sentinels by recognizing pathogens and their pathogen-associated molecular patterns through specific PRRs, such as toll-like receptors (TLRs) and nucleotide-binding oligomerization domain receptors [69].

The activation of PRRs (and particularly TLR-9) on the apical membrane of intraepithelial cells by commensal bacteria results in inhibition of nuclear factor (NF)-kB signaling, activating a process of microbial tolerance [70, 71]. Contact with commensal bacteria via PRRs promotes the production of cytokines, such as thymic stromal lymphopoietin (TSLP), transforming growth factor-β, and retinoic acid, which condition DCs and macrophages toward a tolerogenic phenotype [72, 73]. The CD103+ DCs act as migratory antigen presenting protein by regulating the adaptive immune system, including both T and B lymphocytes. They promote the differentiation of naïve CD4+ T cells into regulatory T cells (FOXP3+) [74, 75]. Furthermore, activated DCs determine, particularly at the level of Peyer’s plaques, the maturation of B cells into IgA-secreting plasma cells [76]. Their induction is also stimulated by additional factors produced by intraepithelial cells, such as the proliferation-inducing ligand (APRIL) and B cell-activating factor (BAFF) [77, 78].

Conversely, the activation of intraepithelial cells PRRs on the basolateral membrane, occurring in case of dysbiosis, intestinal infections, or any damage of the epithelial barrier, promotes the activation of the NF-kB signaling, causing a strong immune response and inflammation [26]. Additionally, DCs can trigger the differentiation of Th1, Th2, and Th17 cells, promoting the development of inflammatory diseases. The intestinal epithelial cells themselves also induce a Th2 response toward helminths and allergens through the production of TSLP and IL-25, eliciting the expansion and differentiation of basophil progenitors and multipotent progenitor cells [79, 80].

Focusing on sIgA, these are localized on the external surface of epithelial cells, and they play multiple roles, such as the protection of the mucosa, the regulation of the microbiota and the modulation of the immune system, preventing bacterium-driven inflammatory, autoimmune, and neoplastic diseases. The spleen plays a major role in the gut immune response through the IgM memory B cells which migrate in the intestinal *lamina propria* and differentiate, through the mechanisms described above, into IgA+ plasma cells. Conditions, such as asplenia and common variable immune deficiency, which are associated with a depletion of circulating IgM memory B cells, are also associated with a defect of sIgA, increasing the risk of gastrointestinal infections [81, 82].

Hence, the alteration of the epithelial barrier, of the immune system and of their intimate interconnection, in association with gut dysbiosis, represents a putative pathogenic mechanism underlying many intestinal and extra-intestinal pathological conditions and a deeper knowledge of this mechanism could provide new therapeutic strategies [83].

## Gut–vascular barrier

The gut–vascular barrier (GVB) is an anatomical structure placed beneath the intestinal epithelium and forms the innermost layer of the intestine wall defense system, hampering, under steady-state conditions, the systemic dissemination of microbes and their toxins through the bloodstream. Yet, it allows the diffusion of nutrients and luminal content (up to a molecular weight of 4 kDa), due to its semipermeable nature, as opposed to less-permissive vascular barriers, such as the brain–blood barrier. Moreover, The GVB enables the migration of immune cells, most importantly CD103^+^ DCs, to mesenteric lymph nodes to mediate tolerogenic responses [84, 85].

**Fig. 3:**
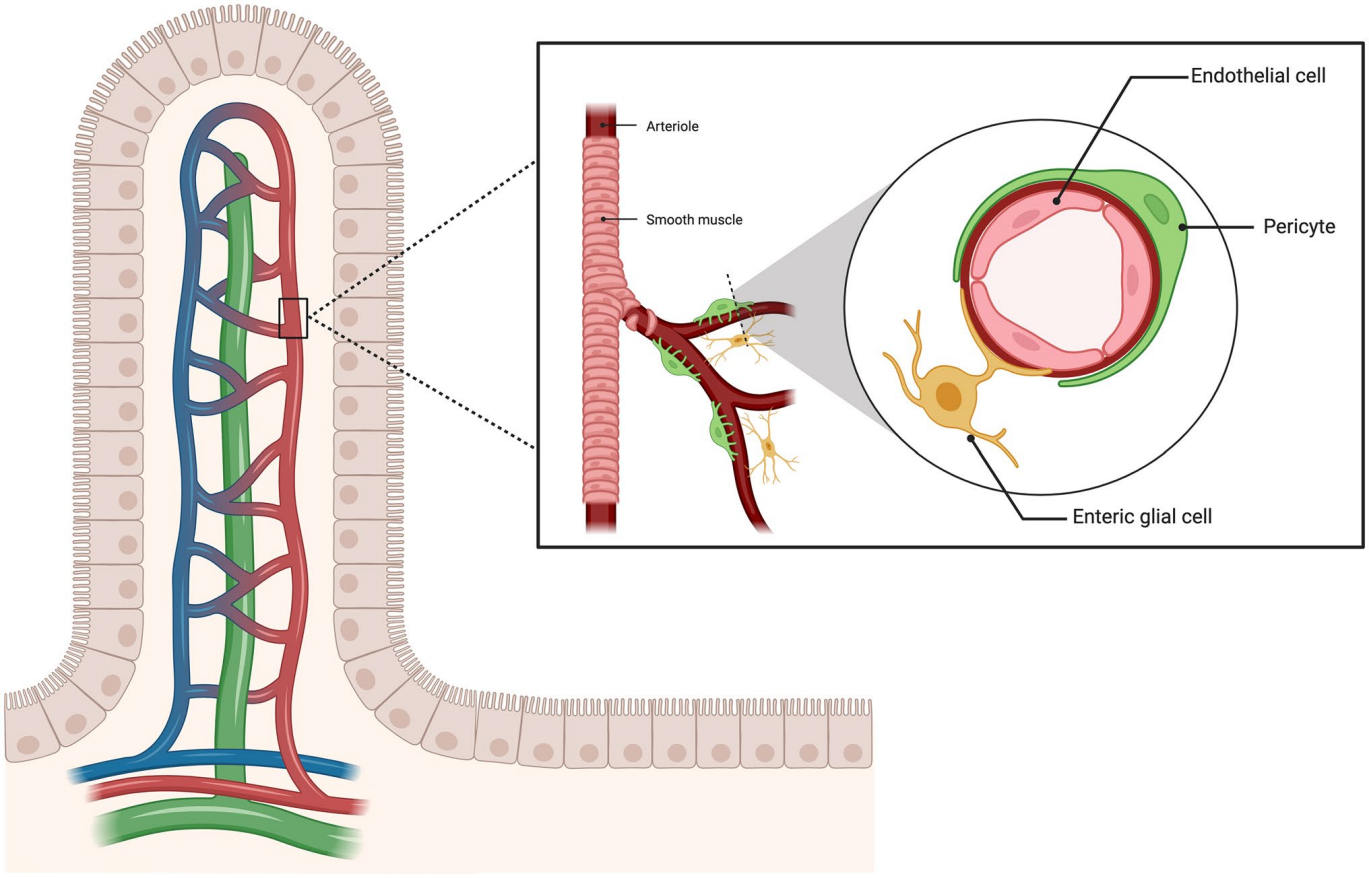
Gut–vascular barrier. The gut–vascular barrier (GVB), placed beneath the intestinal epithelium, forms the inner layer of intestinal defense against microbes dissemination. It is made up of a monolayer of endothelial cells, sealed by adherent and tight junctions, surrounded by pericytes and enteric glial cells. Its semi-permeability is regulated by plasmalemma vesicle protein 1. Created with “BioRender.com”

The GVB, schematically represented in Fig. 3, displays some structural similarities with other vascular barriers, being made up by a monolayer of endothelial cells, which are sealed together by adherent and TJs and surrounded by other specialized accessory cell types, *i.e.,* pericytes and enteric glial cells. The semipermeable nature of the GVB is linked to the fact that the endothelial lining is fenestrated, and the fenestrae are encircled by plasmalemmal vesicle protein 1 (PV-1), a structural element being also present in diaphragms and caveolae [86].

At the molecular level, the integrity of GVB is maintained by the Wnt/beta catenin signaling pathway, whereas PV-1 has a role in basal permeability and can be exploited as a marker of GVB “leakiness” since its increased expression correlates with enhanced vascular permeability [84, 85].

The complex network of cellular components made by endothelial cells, glial cells, and pericytes forms the so-called vascular unit. The contribution of glial cells and pericytes in the formation and maintenance of the GVB is still to be clarified, but these cells are thought to exert relevant supportive functions. More precisely, enteric glial cells contribute to the integrity of the GVB, as attested by the disturbance of its architecture and function, leading to bacterial translocation, which is observed in transgenic murine models deficient of this cell population [87].

Interestingly, it has been recently appreciated that the microbiota, through yet unknown ligands, regulates the dynamics of the GVB targeting the enteric glial cells, and vascular cells, thus having a role on gut angiogenesis and vascular remodeling [84]. Additionally, GVB alterations may lead to the dissemination of dietary antigens, pathogens, and the microbiota and its metabolites to the liver, through the portal vein [85, 88]. More precisely, a mouse model of *Salmonella enterica* gut infection has clarified how the GVB disruption, as assessed by the decreased expression of β-catenin in endothelial cells, paralleled by an increased PV-1 expression, leads to hepatic and splenic dissemination of the infection [89]. Moreover, the GVB has been shown to be dismantled in mouse and humans also in non-infective conditions, *i.e.,* in metabolic diseases, such as alcoholic and non-alcoholic steatohepatitis leading to cirrhosis, in immune-mediated conditions, such as coeliac disease and ankylosing spondylitis, and in cancers, such as the cholangiocarcinoma and colorectal one [89–91].

Interestingly, a bile acid analog, obeticholic acid, similarly to the maintained activation of β-catenin in endothelial cells, was found to stabilize the function of the GVB and to reduce bacterial translocation and intestinal inflammation in a mouse model of cirrhosis [92]. Parallelly, treatment with obeticholic acid has been found to ameliorate the histologic grade of non-alcoholic steatohepatitis in a double-blind, placebo-controlled randomized clinical trial in humans [93].

Taken together, this translational evidence supports the notion that a pivotal pathogenic step in the aforementioned disorders is the architectural and functional alteration of the GVB by the gut microbiota fed with high-fat regimens and by gut inflammation and cancer. Therefore, enhancing the GVB integrity may be promising strategy in the treatment of these disorders.

## Intestinal microbiota

Although the gut microbiota is not a physical barrier, it can still be considered as a functional barrier of the GI tract, constantly interacting with the mucus layer, the epithelium, and the immune system. The term “gut microbiota” refers to the complex and dynamic population of microbes which colonize the entire gut mucosa, and it comprises bacteria, viruses, fungi, and parasites that play a major role in health and homeostasis, having a symbiotic relationship with the host [94, 95].

The gut microbiota is key for maintaining the integrity of the whole intestinal barrier, as shown in several mouse models, even if the mechanisms are not clearly understood. Germ-free mice were found to have immature GI immune responses, as well as smaller abdominal lymph nodes, spleen atrophy, or absence of, decreased IgA-producing plasma cells, and decreased T lymphocytes [96, 97]. This aseptic environment prevents the development of the tolerance toward the symbiotic microbiota and food antigens, the crosstalk with DCs and the epithelium, and the stimulus to the development of TJs. Consequently, the absence of a healthy immune system thus translates into a “leaky gut” syndrome, due to the inefficient GI barrier, leading to weak responses against pathogens, increased bacterial translocation, and death [98].

Similarly, in humans, the gut microbiota is able to shape all immune responses occurring in the gut since birth, throughout life, until death [96]. In fact, since the very first few hours after birth, the microbiota colonizes all GI mucosae, stimulating the tolerance toward “good” bacteria and both self- and certain non-self-antigens, especially those coming with the diet. The bacterial inoculum may happen in different ways depending on the mode of delivery. Physiologically, in the vaginal delivery, the most represented species are *Lactobacillus*, *Prevotella*, or *Sneathia *spp., while in the C-section, *Staphylococcus*, *Corynebacterium*, and *Propionibacterium *spp. predominate [96]. This will constitute the very first microbiota “signature” throughout life. Although the mechanisms are unknown, the microbiota signature occurring in the non-vaginal delivery translates into a greater susceptibility to develop infections early in life [99], and to autoimmune, allergic, and GI diseases in the adulthood [100]. The predisposition to the development of these adverse outcomes has been attributed to an aberrant modulation of the intestinal barriers, especially affecting the mucous production and the permeability, causing a pro-inflammatory environment through the migration of the microbiota and its products to the peripheral circulation. The constant interaction between a healthy microbiota and the mucous is crucial for preventing the disruption of the GI barrier. For example, microbiota-dependent degradation of mucin glycans and the production of shortened glycans may lead to increased susceptibility to bacterial infections and to the development of ulcerative colitis, respectively [101, 102].

Indeed, the microbiota homeostatic balance is influenced by other numerous external and internal factors, spacing from dietary habits (e.g., animal protein consumption induces an increase in *Bacteroides* and *Ruminococcus* and a reduction in *Bifidobacteria* [19]), physical exercise [103], use of antibiotics [104], and many others. Particularly, a low-fiber, high in saturated fat, diet can lead to the disruption of both the mucous layer (which is reduced) and the TJs (which are also reduced), along with a shift to a pro-inflammatory microbiota, producing higher levels of TNF-alfa, IL-6, and IL-1 [105]. Microbiota-driven gut leakiness has also been implicated in the development of non-GI disorders, namely Parkinson’s disease, Alzheimer’s disease, and frailty [106, 107]. An antioxidant diet may counteract these detrimental effects, being able to increase fiber-fermenting and butyrate-producing bacteria, such as the family *Ruminococcaceae* and the members of the genus *Faecalibacterium*, determining a decrease of zonulin expression, and thus a tighter intestinal barrier [108, 109]. Consistently, the manipulation of the gut microbiota through *Bifidobacterium adolescentis* and *Bifidobacterium lactis* was found to reduce intestinal permeability in humans [110, 111].

Finally, some insights regarding the interaction between the microbiota and the other intestinal barriers derive from studies conducted in immune-mediated GI diseases, such as coeliac disease and inflammatory bowel disease. Regarding coeliac disease, there is a large amount of literature, which is often heterogeneous and contradictory. To summarize, an increase in the amounts of Gram-negative genera, including *Bacteroides*, *Prevotella*, and *Escherichia*, and reduced amounts of the anti-inflammatory genera *Bifidobacteria* and *Lactobacilli* have been found, although their significance in determining the disease is uncertain [112, 113]. Similarly to what happens in mice [96], it has been hypothesized that spleen hypofunction in coeliac disease may be due to a disruption of the intestinal barrier, also including an altered gut microbiota, but no form evidence is available [82]. In inflammatory bowel disease, a perturbation of the microbiota clearly emerges from the current literature, but whether this is primary or secondary to the inflammation is unknown. Patients with active inflammatory bowel disease have a lower abundance of *Clostridium coccoides*, *Clostridium leptum*, *Faecalibacterium prausnitzii*, and *Bifidobacterium* compared to remission, and patients with active CD have fewer *C. leptum*, *F. prausnitzii*, and *Bifidobacterium*, but not *C. coccoides* [114]. Moreover, levels of *Bacteroides* tend to be lower in patients with IBD compared to the general population [115]. Because of the aforementioned literature, interventional studies both on microbiota transplantation and probiotics supplementation are emerging [116, 117]; however, studies looking at intestinal barrier as a target of microbiota manipulation are completely lacking.

## Conclusion: a unifying view of the intestinal barriers

We have herein described in a narrative fashion the different components of the intestinal barriers, by dissecting the state-of-art about the physiology and regulation of each component, namely the epithelial barrier, the mucosal immune system, the gut–vascular barrier, and the gut microbiota. However, this is a clear reductionist view of the matter, as in vivo a clear anatomical and functional distinction cannot be made, and all these structures are mutually influenced by each other. To summarize, the first immunological imprinting to the human intestinal barrier is provided by the interaction of the microbiota and the mucosal immune system. Although the fine mechanisms are unknown, this process is thought to be the key to promoting the basilar immune tolerance and the development of the local and systemic immunity. The interaction between microbe surface proteins and the DCs promote the production of IL-10, a tolerogenic cytokine. The epithelial cells, under this physiological stimulus, are responsible for the production of AJs and TJs, which regulate the optimal permeability and hence may filter or amplify local and systemic immune responses. A further “gate” that regulates this function is the GVB, which is called into question for a more precise and fine response to local and systemic inflammation. Indeed, this tentative unifying view is just speculative and derives from the current available knowledge, which needs to be confirmed by ad hoc studies. Some derivative knowledge comes from GI disease models, although uncertainty still exists around the primary “hit” that determines the disruption of the barrier, or whether the barrier alteration is the *primum movens* triggering the disease.

## Outlook

Significant progress has been made about the knowledge of the gut barrier composition and the anatomical and immunological mechanisms underlying the interaction between this barrier and the microbiota. Since the interaction among all the discussed components seems to play a key role in the pathogenesis of inflammatory diseases, both GI and non-GI, their regulation represents a challenge. In the future, the use of molecules capable of maintaining or restoring the function of the intestinal barrier and its physiological interactions with the microbiota could represent a chance in the prevention and treatment of these diseases, also by promoting healthy aging. Before reaching this ambitious goal, studies looking at the intestinal barrier as a whole in humans are eagerly awaited.

## Data Availability

Not applicable, as no original data are reported.
